# Field Validation of the Southern Rock Lobster Paralytic Shellfish Toxin Monitoring Program in Tasmania, Australia

**DOI:** 10.3390/md19090510

**Published:** 2021-09-08

**Authors:** Alison Turnbull, Juan José Dorantes-Aranda, Tom Madigan, Jessica Jolley, Hilary Revill, Tim Harwood, Gustaaf Hallegraeff

**Affiliations:** 1 Institute of Marine and Antarctic Studies, University of Tasmania, Taroona, TAS 7053, Australia; juanjodorantes@gmail.com (J.J.D.-A.); Gustaaf.hallegraeff@utas.edu.au (G.H.); 2 South Australia Research and Development Institute, Urrbrae, SA 5064, Australia; madigan.tom@hotmail.com (T.M.); jessica.jolley@sa.gov.au (J.J.); 3 Department of Primary Industry, Parks, Water and Environment, Hobart, TAS 7001, Australia; hilary.revill@dpipwe.tas.gov.au; 4 Cawthron Institute, Nelson 7010, New Zealand; tim.harwood@cawthron.org.nz

**Keywords:** marine biotoxin, non-traditional vector, *Jasus edwardsii*, lobster, uptake, depuration, risk management

## Abstract

Paralytic shellfish toxins (PST) are found in the hepatopancreas of Southern Rock Lobster *Jasus edwardsii* from the east coast of Tasmania in association with blooms of the toxic dinoflagellate *Alexandrium catenella*. Tasmania’s rock lobster fishery is one of the state’s most important wild capture fisheries, supporting a significant commercial industry (AUD 97M) and recreational fishing sector. A comprehensive 8 years of field data collected across multiple sites has allowed continued improvements to the risk management program protecting public health and market access for the Tasmanian lobster fishery. High variability was seen in toxin levels between individuals, sites, months, and years. The highest risk sites were those on the central east coast, with July to January identified as the most at-risk months. Relatively high uptake rates were observed (exponential rate of 2% per day), similar to filter-feeding mussels, and meant that lobster accumulated toxins quickly. Similarly, lobsters were relatively fast detoxifiers, losing up to 3% PST per day, following bloom demise. Mussel sentinel lines were effective in indicating a risk of elevated PST in lobster hepatopancreas, with annual baseline monitoring costing approximately 0.06% of the industry value. In addition, it was determined that if the mean hepatopancreas PST levels in five individual lobsters from a site were <0.22 mg STX equiv. kg^−1^, there is a 97.5% probability that any lobster from that site would be below the bivalve maximum level of 0.8 mg STX equiv. kg^−1^. The combination of using a sentinel species to identify risk areas and sampling five individual lobsters at a particular site, provides a cost-effective strategy for managing PST risk in the Tasmanian commercial lobster fishery.

## 1. Introduction

Recurrent blooms of the toxic dinoflagellate *A. catenella* (Whedon and Kofoid) Balech have occurred in winter on the east coast of Tasmania since 2012 [1]. The species involved is highly toxic, resulting in paralytic shellfish toxins (PST) increasing rapidly in bivalve molluscs. Public health and market access risks are managed through weekly monitoring of toxin levels in shellfish flesh, with closures occurring when PST exceeds the domestic regulatory maximum level (ML) of 0.8 mg saxitoxin (STX) equiv. kg^−1^ in bivalve flesh. Closures may be for extended periods (over 20 weeks; unpublished data), with the maximum PST level detected of 150 mg STX equiv. kg^−1^ in mussels in 2017 grossly exceeding the ML [1].

During the initial bloom in 2012, PST was detected in multiple bivalve species (oysters, mussels, scallops, and clams) and several non-traditional vectors (rock lobsters, abalone, and giant crab) [2]. Close to 30% of the rock lobster hepatopancreas samples (*n* = 93) collected during the bloom exceeded the domestic bivalve ML.

Following the 2012 bloom and these unexpected PST results, industry, regulators, and scientific experts conducted a risk ranking for all commercial seafood species in Tasmania [3]. The risk ranking process included consideration of commercial catch volumes for all seafood, edible portions of the seafood, ecological groups and trophic levels in the food web, the four main marine biotoxin groups (paralytic, diarrhetic, amnesic and neurotoxic shellfish toxins) and their history in Tasmania, and peer-reviewed literature for toxin accumulation in non-traditional vectors worldwide. The review concluded that the toxin: species pairing of PST in Southern Rock Lobster (*Jasus edwarsdii* Hutton) represented the greatest food/hazard risk, warranting a specific risk management program. Tasmania’s rock lobster fishery is one of the state’s most important wild capture fisheries, supporting a significant commercial industry (AUD 97M) and recreational fishing sector.

A significant body of research has occurred to underpin the Tasmanian rock lobster risk management program. Toxins were confirmed to accumulate to levels of concern only in the lobster hepatopancreas [4]. Cooking studies demonstrated that the toxicity of the hepatopancreas remained the same during steaming and boiling, with no change in the toxin profile but a reduction in the amount of hepatopancreas present for consumption [5]. A survey of recreational fishers in Tasmania and South Australia confirmed that the hepatopancreas was commonly consumed [6]. Consumer exposure to PST was estimated using a 2-D Monte Carlo model [7]. The exposure assessment concluded that lobster hepatopancreas consumption during *A. catenella* blooms may be concerning for a small proportion of consumers but instigating harvesting restrictions for lobsters when levels exceed the bivalve ML reduced the probability of illness occurring.

Environmental conditions likely to trigger *A. catenella* blooms were determined to be water temperatures of 10–15 °C and stratification of coastal waters via salinity and/or temperature gradients [1]. Experimental studies feeding highly toxic mussels to *J. edwardsii* found: exponential uptake and depuration rates of 6 and 7% per day, respectively; potential excretion routes for PST are via the faeces, antennal glands and gill; there is no impact of PST on lobster health; and confirmed PST uptake did not occur through direct exposure to *A. catenella* cells, as would occur in boat wells, sea cages and holding facilities of the live lobster supply chain [8,9,10].

**Figure 1:**
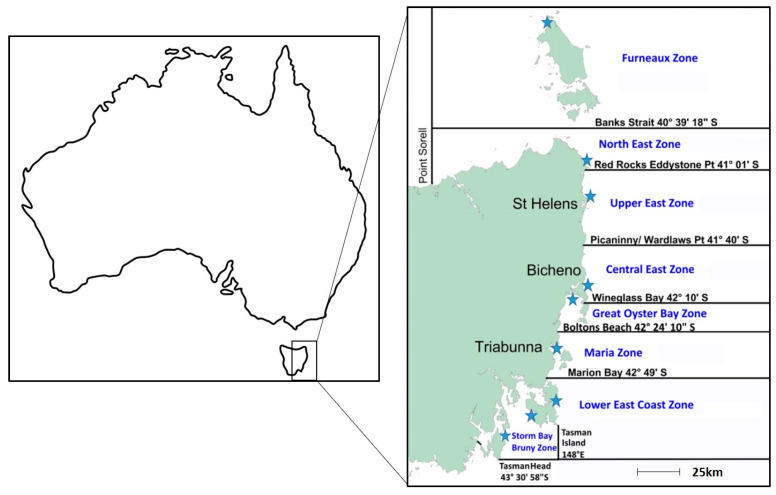
Tasmanian biotoxin management zones for *J. edwardsii*, as provided in the Rock Lobster Biotoxin Monitoring and Decision Protocols 2020 [11]. Blue stars indicate the location of sentinel mussel sites.

The current risk management of biotoxins in Tasmanian lobsters is described in the Rock Lobster Biotoxin Monitoring Plan [11]. The east coast of Tasmania is divided into eight rock lobster biotoxin management zones, as depicted in Figure 1. Biotoxin sampling in lobster is triggered during high-risk seasons (winter–spring) by elevated PST levels in the common blue mussel *Mytilus galloprovincialis* Lamark from sentinel mussel sites. Management zones are closed for lobster harvest prior to sampling and remained closed until biotoxin results indicate it is safe to harvest. The closure for sampling (including research sampling) is to avoid the risk of a non-compliant lobster entering the export market chain of supply. The industry currently has little traceability capacity: if there was to be a market recall due to a non-compliant lobster PST sample, it could impact all Southern Rock Lobster harvested between the date of sampling and receipt of the PST results. Previously, the average time between sampling and receipt of toxin results was 7–10 days; however, the adoption of the LC-MS/MS technique [12,13] in Tasmania has reduced this to an average of 3 days.

The sampling closures are formal legislative closures with significant penalties. Fishers are notified at least 48 h ahead of intended sampling date. Commercial fishing cannot resume until the PST results have been received, reviewed against the decision protocols and the zone formally reopened. There is a high level of industry awareness and support for the biotoxin closure policy.

Risk management sampling involves harvesting five legal sized lobster from one site within each zone and individually analysing each hepatopancreas for PST. Zones are deemed safe for harvest if all hepatopancreas samples contain <0.5 mg STX equiv. kg^−1^, unsafe for harvest if any hepatopancreas sample exceeds 0.8 mg STX equiv. kg^−1^, and questionable if any hepatopancreas sample is between 0.5 and 0.8 mg STX equiv. kg^−1^. In the latter case, further information is sought to allow an assessment of the risk. Management zones may also be closed for fisheries management reasons during the high-risk period, with seasonal fisheries closures usually occurring from early October to late November/early December for the upper zones, and from early September to late November/early December for the remaining zones.

Field studies of PST in *J. edwardsii* on the east coast of Tasmania occurred concurrently with the experimental work described above from 2012 to 2020 inclusive. The studies aimed to identify field accumulation and depuration rates, and variability in PST accumulation in lobster individuals to produce data that, combined with the experimental studies, could improve risk management activities. The field studies aimed to refine information on high-risk months and sites on the east coast of Tasmania, inform sampling frequency and the minimum number of representative samples, and evaluate the use of sentinel species as a risk management option.

## 2. Results

All PST results reported herein are expressed in mg STX equiv. kg^−1^ in line with the current Australian regulatory requirement for PST in bivalve shellfish and also used for PST risk management in Southern Rock Lobster. Furthermore, this aligns with the ML for China [14], the main export destination for Tasmanian lobster [15]. The authors point out that the Codex reporting units are different (mg STX.2HCl equiv. kg^−1^), and our previous research on PST in Southern Rock Lobster has been reported following Codex guidelines (producing results that are effectively 24% higher [14]).

### 2.1. Variation in PST Levels across Sites and Time

**Figure 2:**
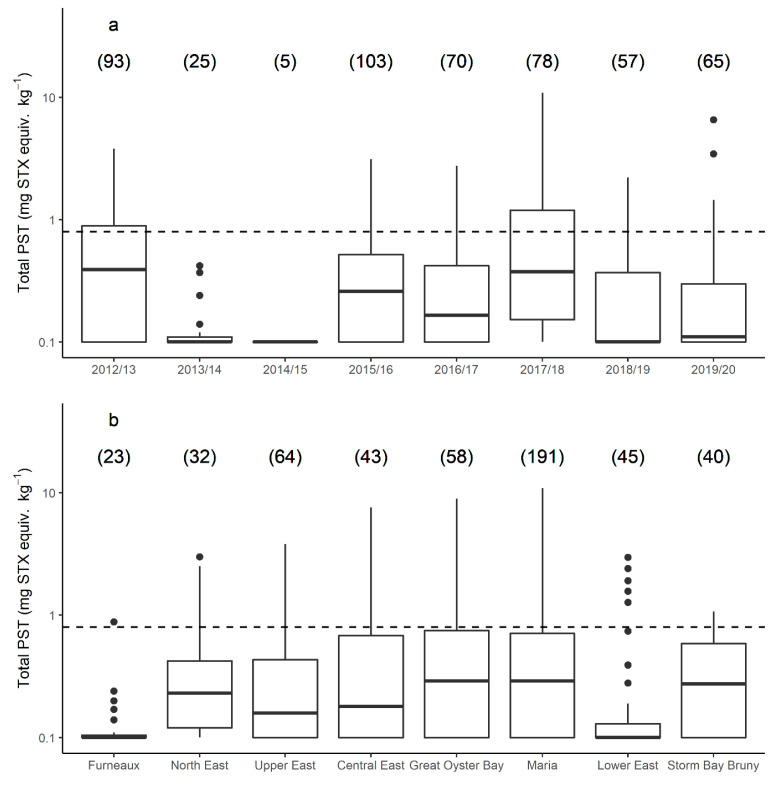
Paralytic shellfish toxins (log scale) in *J. edwardsii* hepatopancreas across all bloom years (**a**) and management zones (**b**) on the east coast of Tasmania from 2012 to 2000. Bloom years are from May until April each year: management zones relate to those given in Figure 1, presented (left to right) from north to south. Boxes indicate median, first and third quartiles; whiskers extend to maximum of 1.5 times the interquartile range; outlying points are plotted individually; number of samples in brackets. Bivalve maximum allowable level shown as black dotted line.

Recurrent blooms of *A. catenella* occurred during the 8 year study period, triggering risk monitoring and research sampling in all years. Of the 496 *J. edwardsii* hepatopancreas samples analysed for PST between 2012 and 2020, 100 (20%) did not contain PST above the level of reporting. Toxin accumulation in lobster > 0.8 mg STX equiv. kg^−1^ occurred in all years, except the 2013/2014 and 2014/2015 bloom years (Figure 2a), with 86 (17%) lobster exceeding this level (Figure 2). There was a significant difference between years with respect to the level of PST accumulated (*p* value < 0.005). The length and timing of the toxin event also varied annually. The longest biotoxin-related harvest closure for lobster fishing occurred in the Maria zone during 2015/2016, lasting 14 weeks. With the exception of one closure in the southern zone of Storm Bay Bruny in April 2013, the earliest month that lobster hepatopancreas first exceeded the bivalve ML was July (Great Oyster Bay zone, 2016), and the latest was November (several sites, several years), although it should be noted that sampling often did not occur in October when zones were closed for fisheries management reasons.

**Figure 3:**
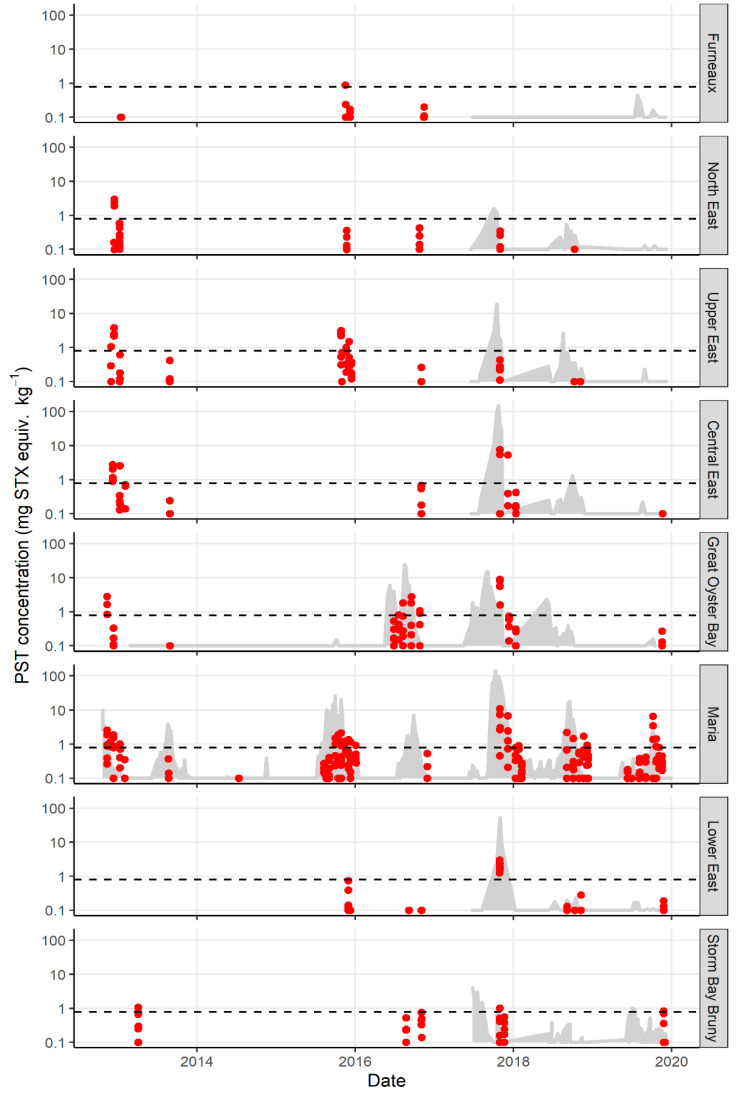
Paralytic shellfish toxins (log scale) in all management zones on the east coast of Tasmania from 2012 to 2020. Bivalve ML shown as black dotted line. PST concentration in *J. edwardsii* hepatopancreas (red dots) exceeds the bivalve ML in every zone on at least one occasion. Prior to 2017, *M. galloprovincialis* sentinel monitoring (grey shaded area) only occurred in Great Oyster Bay and Maria zones. PST in sentinel mussels exceed the bivalve ML prior to lobster hepatopancreas on all occasions except in Storm Bay Bruny in 2019.

There was a significant difference in the total PST accumulated in each zone (*p* value 0.018), with the highest PST accumulation occurring in the central zones of Central East, Great Oyster Bay, and Maria (Figure 2b). The maximum total PST level recorded in lobster hepatopancreas was 10.9 mg STX equiv. kg^−1^ from the Maria zone in 2017. The northern zones of Furneaux and North East each only exceeded 0.8 mg STX equiv. kg^−1^ in one year late in the season (October 2015 and November 2012 respectively, Figure 3). Similarly, lobster hepatopancreas exceedances of the bivalve ML in the southern end of the east coast were more sporadic, with lower maximum PST levels and a shorter period of contamination.

### 2.2. Variation in PST Accumulation within Sites

There was a high degree of variability in PST accumulation in individual lobsters harvested from one site on the same date (Figure 3; the coefficient of variation ranged from 0.01 to 1.80, averaging 0.79 when the mean PST > 0.1 mg STX equiv. kg^−1^). Toxin accumulation was not significantly different between sexes (*p* value 0.51) and was not related to carapace length or weight (*p* values 0.78 and 0.43, respectively).

**Figure 4:**
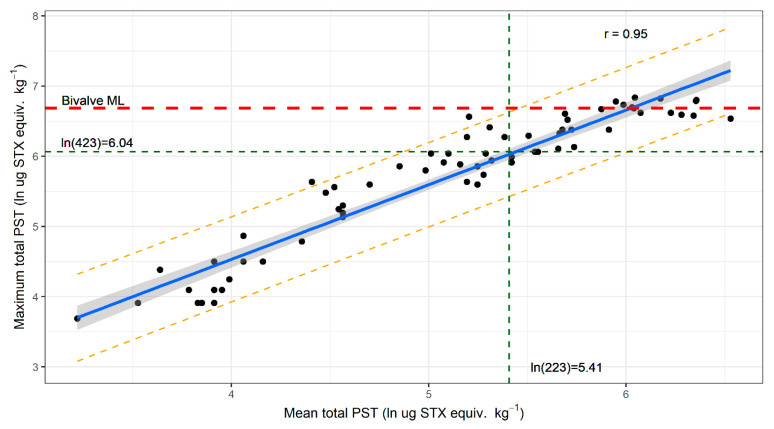
Linear model of the mean and maximum total PST concentrations found in *J. edwardsii* hepatopancreas at each sample event at the start and end of *A. catenella* blooms on the east coast of Tasmania between 2012 and 2019 (black dots, *n* = 68), showing the best fit regression line (blue), 95% confidence interval (dark grey shading) and 95% prediction interval (orange dotted line). The intersection of the bivalve maximum level (dashed red line) with the upper 95% prediction interval allows interpolation (green dotted lines) of the mean total PST concentration that gives 97.5% probability that the lobster population at a site is below the bivalve ML.

To determine if the regulatory sampling regime adequately represented PST levels across the lobster population of a site with respect to the bivalve ML, data from the start and end of the blooms (defined as periods when all samples from a site were <1 mg STX equiv. kg^−1^) were examined. This subset of data was chosen because bloom initiation and termination when toxin levels are below the bivalve ML is the period of most concern to the risk management program and the consistency of variability across the entire bloom was unknown. The PST concentrations in the hepatopancreas in both data sets were normal and homogeneous when transformed using a log function. The total PST levels from the two data sets were not significantly different (*p* value 0.47); therefore, the data were combined for analysis. The mean and the maximum PST concentration from each sampling event were linearly related (Figure 4; r = 0.95). Interpolation of the upper 95% prediction interval intersection with the bivalve ML was then used to obtain the estimates for the mean PST of a sample event, below which there was 97.5% or greater probability that the population of that site would comply with the bivalve ML. Figure 4 demonstrates that this level of confidence would be achieved when the mean PST concentration at a site was at or below 0.22 mg STX equiv. kg^−1^. On average, this occurs when the maximum PST concentration is at or below 0.42 mg STX equiv. kg^−1^.

### 2.3. Mussels as a Sentinel Species

Mussels were first investigated as a sentinel species for lobster management zones during the research sampling in 2015. Mussel lines were extended to all management zones where commercial bivalve aquaculture was not occurring on the coast in 2017. Mussel accumulation of PST over a bloom season rose and fell rapidly, with high levels of PST providing warning of PST risk in lobster (Figure 3). For sites with matched *J. edwardsii* and *M. galloprovincialis* data, nine exceedances of the bivalve ML in *J. edwardsii* hepatopancreas were recorded. All except one followed mussel exceedances. The only event that was not predicted by prior accumulation of toxins in mussels was Storm Bay Bruny in November 2019. Mussels exceeded the bivalve ML between 6 days (Maria zone October 2019) and 3 months (Great Oyster Bay zone October 2017) prior to lobster hepatopancreas exceedance, although zones that were closed for fisheries management reasons were not sampled until the fisheries closure was nearly over.

### 2.4. Field PST Accumulation and Depuration Rates of PST

Field uptake and depuration rates for total PST in *J. edwardsii*, calculated from linear models of log PST on the occasions when at least four sampling events took place at one site during the period of interest, are given in Table 1. Rates could only be calculated for research sampling in the Maria zone during the 2015/2016 and 2017/2018 bloom years for uptake and the 2012/2013, 2017/2018 and 2019/2020 bloom years for depuration. Corresponding rates of PST uptake and depuration for *M. galloprovincialis* at sentinel sites over the same period were similar (but higher) to those observed for lobsters. The only occasion where rates were significantly different between the two species was depuration during the 2017/2018 bloom. This was a particularly dense bloom, where mussel toxicity reached 150 mg STX equiv. kg^−1^. Residual variances in the rates of uptake and depuration are larger for lobster than mussels, reflecting the high variability of PST concentrations between individuals sampled on each occasion.

### 2.5. The Interaction of Fisheries and Biotoxin Closures

**Figure 5:**
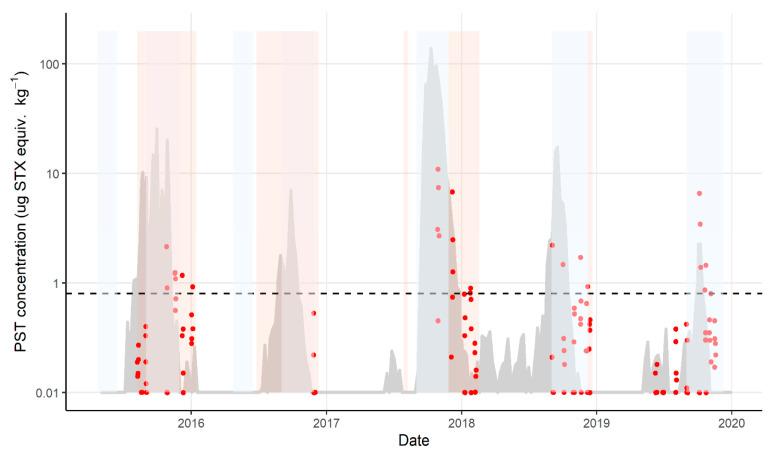
Paralytic shellfish toxins concentrations in *J. edwardsii* and *M. galloprovincialis*, Maria zone, from 2015 to 2020: *J. edwardsii* hepatopancreas (red dots); *M. galloprovincialis* (grey shaded area, screen and confirmed data); fisheries management closures shaded blue and lobster biotoxin closures shaded orange. Horizontal dotted line represents the bivalve ML.

The seasonal fisheries management zone closures occur on the Tasmanian east coast for reasons such as lobster resilience at the time of moulting, handling of berried females, and catch constraint and resource sharing between the recreational and commercial sectors. These closures occur in the peak biotoxin season (Figure 5). Mussel sentinel monitoring continues through these closures, with risk management sampling of lobsters occurring prior to re-opening if any biotoxin activity has been detected. At the highest PST risk zone, Maria, every lobster exceedance since 2015 has begun during a fisheries closure.

### 2.6. PST Profiles

**Figure 6:**
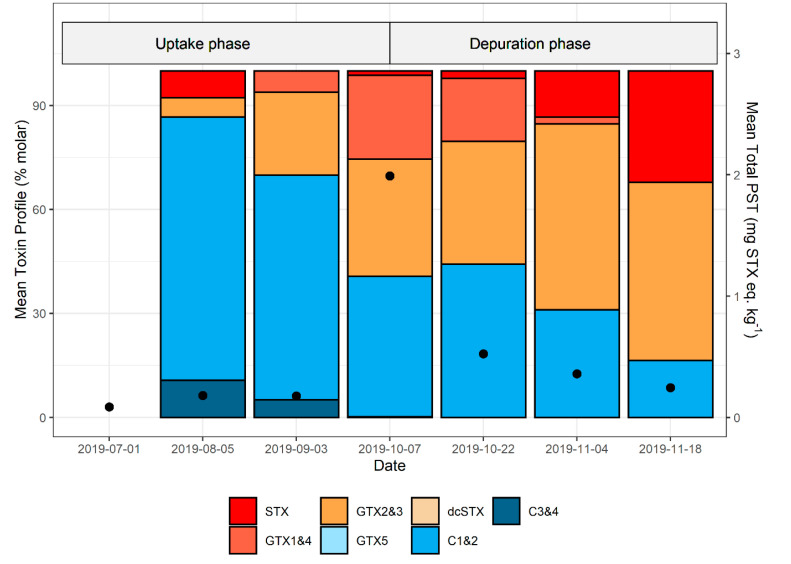
Mean paralytic shellfish toxin profiles (molar percentage) and mean total PST (toxicity equivalents) in *J. edwardsii* hepatopancreas from Maria Island during the 2019/2020 bloom. Toxin profile data only includes lobster with total PST > 0.1 mg STX equiv. kg^−1^, whilst total PST data includes all samples taken on that day. Mean total PST shown as black dots.

The molar PST profile of *J. edswardsii* hepatopancreas was generally dominated by gonyautoxins (GTX)1&4, GTX2&3, and the N-sulfocarbmoyl toxins C1&2, with minor percentages of STX, C3&4, and decarbomoyl saxitoxin (dcSTX). Occasionally, neosaxitoxin (NEO) or decarbomoyl gonyautoxin (dcGTX)2&3 were present above their respective reporting limits. The same analogues were found in *M. galloprovinciallis*, with GTX1&4, GTX2&3, and C1&2 also dominant in this shellfish species. The molar percentage contribution of analogues from samples taken from the same site at the same time were variable, particularly with respect to the amount of STX present, which ranged from 0 to 100% during some events. To examine changes in PST profiles during uptake and depuration across one bloom, the average molar percent profiles of each sample event from Maria 2019/20 bloom were investigated (Figure 6; see Supplementary Table S1 for individual profiles in millimoles). This was the only bloom period where both uptake and depuration rates were able to be calculated. PST levels in lobster on the first sample event were low (<0.2 mg STX equiv. kg^−1^). On the second sample event, close to 90% of the PST analogues present were C toxins, the majority of which were C1&2. During both the uptake and depuration phases, the mean percentage of C toxins consistently decreased (Figure 6), whilst GTX2&3 increased. There was a decrease in GTX1&4 and a concomitant increase in STX during depuration.

## 3. Discussion

Risk management and research sampling of *J. edwardsii* hepatopancreas from the east coast of Tasmania only occurred in association with blooms of *A. catenella.* As a result, hepatopancreas samples frequently contained PST, often at concentrations above bivalve ML. A high level of variability was seen between bloom years with respect to the site, timing, and level of PST accumulation. Toxicity was greatest in the centre of the east coast with exceedances of the bivalve ML occurring during the months of July to January. The one exception to this was an exceedance in the Storm Bay Bruny zone in April 2013. This management zone includes the mouth of the Derwent Estuary, where *Gymnodinium catenatum* frequently blooms, particularly during spring and autumn [16,17]. Whilst no data is available for phytoplankton assemblages in the area during April 2013, this is a plausible PST source.

The high variability between individuals concurrently collected from one site is likely to be caused in part by variations in amount of PST consumed. This is influence by both the feeding rate of each lobster and the total PST amount in each prey item. Lobster feed on a wide variety of prey items, including molluscs, small crustaceans, echinoderms, and other benthic invertebrates [18,19], which can be expected to contain highly variable amounts of toxin due to their different feeding strategies.

The variability between PST concentrations in individual lobster creates difficulties in risk management decisions for the total lobster population within each zone. Risk management procedures need to be practical and affordable. The significant data set collected here has allowed an estimate of population risk of exceedance of the bivalve ML associated with the collection of five animals. Five animals represent an achievable sampling effort at an analytical cost of approximately AUD 1250-3000, depending on the PST analytical technique used, the analysing laboratory and the turnaround time requested. When the mean PST concentration of five lobster from a site is less than 0.22 mg STX equiv. kg^−1^, there is a 97.5% probability that the population at that site will be below the bivalve ML when harvesting is occurring. A less conservative approach could be taken if more lobsters were sampled on each occasion, but this would require a greater sampling effort and analytic expense to achieve the same level of confidence.

Crustacean biotoxin monitoring programs in other countries also state the need to test multiple animals from a site to determine toxicity and may use conservative triggers for closure. Examples include *J. edwardsii* monitoring in New Zealand [20] and Dungeness crab monitoring in California [21,22]. Both the New Zealand and Californian programs also monitor toxins in bivalve harvest areas and draw on data from phytoplankton monitoring to assist in their risk management activities. Other crustacean risk management programs rely on toxicity testing of product prior to export [23].

In bivalve biotoxin risk management programs, phytoplankton are often used as a predictive management tool [24]. However, *A. catenella* on the Tasmanian east coast has proven to be highly toxic [25], and as a result, toxin accumulation in bivalve shellfish has exceeded regulatory limits on several occasions prior to, or concurrently with, toxic cells being identified in the water column. Thus, in Tasmania, more emphasis is placed on weekly bivalve biotoxin testing supported by monthly phytoplankton monitoring to confirm toxic species.

Mussels were first investigated as a sentinel species for lobster risk management by Madigan et al. in the initial research project following the 2012 bloom [26]. Mussels make ideal sentinels as their high feeding filtration rates result in rapid toxin accumulation, they provide an integrated toxicity estimate (compared to a single point in time as offered by a phytoplankton sample), analytical methods are well developed for this matrix, and they are easy to collect off existing marine infrastructure, or lines are easy to deploy where no infrastructure exists. Sentinel mussel sites have since been installed in all zones where commercial bivalve aquaculture is not occurring on the coast.

There have been nine harvest closures instigated for *J. edwardsii* in areas where mussel sites were concurrently sampled with lobster. All except one of these closures was preceded by elevated toxicity in the mussels. The exception was Storm Bay Bruny during November 2019. In this case, the sample sites for mussels and lobsters were not in proximity. This raises the issue of variability at different sites within zones: variability is acknowledged to be likely but has not yet been investigated in detail.

Experimental data have shown that *J. edwardsii* can accumulate PST in the hepatopancreas to over the bivalve ML within 4 days if fed highly toxic mussels in large quantities [8]. The exponential uptake rate for that study over a period of 27 days was 6% per day. Field rates of PST accumulation in *J. edwardsii* hepatopancreas in this study were similar (1 and 2% per day), indicating lobster were feeding on PST-rich prey or consuming large volumes of prey containing some PST.

The depuration rates determined for lobster in this field study varied between 1.7 and 3.1% per day, similar to the 2 and 7% found in experimental studies of PST depuration from fed *J. edwardsii* [4,8]. Depuration during experimental studies occurred when no toxic feed was present, resulting in immediate depuration at maximum rates for the conditions studied. The persistence of PST at high levels in lobster in the field after mussel toxicity has declined, as shown in Figure 5, suggests that at least some of the prey items are retaining PST longer than the mussels. However, the similarity of field and experimental depuration rates suggests that toxin retention in most prey items is not long-term. The data confirms that *J. edwardsii* in the environment clear PST from their hepatopancreas relatively quickly once a toxin event has passed. From the field depuration rates measured here, it would take lobsters 15–27 days for depuration from twice the bivalve regulatory level to be below the bivalve regulatory level. In all the field events studied, depuration to the bivalve ML occurred in mussels prior to lobsters, demonstrating that the usefulness of the mussel sentinel lines is valid for both uptake and depuration.

The PST analogues present in *J. edwardsii* hepatopancreas were the same as those seen in experimental studies using mussels from Tasmanian east coast events as the prey source [4,8]. In the Maria zone in 2019, the proportion of C1&2 and C3&4 were higher than seen in the experimental studies, likely due to the prey sources available in the field. All C toxins decreased as the bloom progressed through uptake and depuration phases. In the experimental studies, this decrease was only seen during depuration. In both the field and experimental studies GTX1&4 reduced during depuration whilst the proportion of GTX2&3 increased. A similar pattern was observed with the field samples, with STX also increasing during depuration. A reduction of GTX1&4 relative to GTX2&3 was also described in the spiny lobster *Panulirus stimpsoni* during depuration [27].

The risk management of PST in Tasmanian *J. edwardsii* has evolved since the first detection of PST associated with an *A. catenella* bloom in 2012, in response to research outputs that have improved the risk-based response. A key objective of the rock lobster biotoxin monitoring program is to ensure that the risk of a Tasmanian rock lobster exceeding any import standard is as low as possible. China is an important market destination for this high value fishery and has a PST standard that applies to all seafood. Thus, using the bivalve regulatory ML, which is the same as the Chinese regulatory ML, serves the dual purpose of protecting both public health [7] and market access. There have been no illnesses reported in association with PST in Tasmanian lobster hepatopancreas.

The use of mussel lines to provide an easy to access a source of bivalve shellfish for PST testing in zones where wild or aquaculture shellfish are not abundant or accessible has been a significant improvement to extend the capacity to sample areas of relevance/importance to the rock lobster fishery. These sentinel bivalve shellfish act as an early warning system, triggering PST testing in lobsters. The two-tier monitoring program reduces both the expense of lobster sample collection and analysis, and the cost to industry of lost fishing opportunity through zone closures enacted prior to sampling.

Overlaying the biotoxin closures are regional seasonal management closures. These are implemented each year for a variety of reasons, including: lobster resilience at time of moulting, handling of berried females, and catch constraint and resource sharing between the recreational and commercial sectors. The majority of the east coast is under a regional seasonal closure between 1 September and early December. Bivalve sentinel PST testing continues throughout this seasonal closure period to inform whether lobster sampling is required prior to the scheduled reopening of the east coast fishery. For this reason, the first lobster biotoxin samples are often not taken until mid/late November when a bloom event may have passed its peak.

Biotoxin zone closures, particularly if multiple zones are closed for an extended period of time, can have significant fishery management impacts through the concentration of fishing effort in remaining open part of the fishery. To reduce the risk of localised stock depletion, a biotoxin zone may remain closed even though the lobster PST results meet the re-opening decision criteria.

The cost of the monitoring program (sample collection and analysis) is shared between rock lobster licence holders and the Tasmanian Government. The baseline monitoring of PST in sentinel bivalves costs approximately AUD 60,000 per annum (0.06% of Tasmanian lobster industry value), with monitoring costs increasing when bloom activity is detected, triggering increased sampling frequencies and sampling and analysis of lobster. The relatively low cost of monitoring bivalve sentinel PST data to provide an early warning of bloom activity along the whole eastern region of Tasmanian is a critical component for a cost-effective program, given the unpredictable spatial and temporal distribution/occurrence of *A. catenella* blooms.

This study represents the first comprehensive field study published on PST accumulation in lobster. Risk management programs addressing PST in lobster exist in other countries [28], supported by experimental work [27,29,30,31,32,33]. However, no field studies have been published. For the Tasmanian rock lobster fishery, the combination of previously published experimental work [4,5,6,7,8,9,10,34] and the field studies reported herein have provided a strong evidence base for continued progressive improvements to the Tasmanian risk management program.

## 4. Materials and Methods

### 4.1. Monitoring of PST in J. edwardsii Hepatopancreas

Field sampling of *J. edwardsii* occurred either on a regular basis through the high-risk period (research sampling; Great Oyster Bay and Maria zones) or when triggered from mussel sentinel results (risk management sampling in all zones [11]). In both cases, lobster (*n* = 5) were caught at each site, normally by SCUBA divers in <8 m, but occasionally from lobster traps. Lobster were kept on ice and transported back to the laboratory, usually within 24 h. Lobster where then euthanized, sexed, weighed, carapace length measured and dissected to remove the hepatopancreas. Each lobster hepatopancreas was homogenized and individually analysed for PST. In some risk management samples prior to 2017, ovaries were included in the hepatopancreas sample. Hepatopancreas samples were either transported immediately to Analytical Services Tasmania or Symbio for PST analysis as above, or frozen at −40 °C and transported to Cawthron Institute New Zealand for analysis.

### 4.2. Mussel Sentinel Sampling

At least one sentinel mussel site exists in each lobster biotoxin management zone. These may be either commercial marine farms in coastal areas, marine infrastructure in coastal areas (e.g., jetties), or specific mooring lines with bags of *M. galloprovincialis* attached prior to the beginning of each risk season.

Pooled samples of mussels (*n* = 15) from each site were homogenised on a weekly (bivalve commercial monitoring program) or fortnightly (lobster mussel sentinel program) basis and analysed for PST at Symbio (prior to December 2017) or Analytical Services Tasmania (post November 2017). Occasionally, only PST screen analysis was undertaken (Section 4.4). The frequency of the sentinel sampling increased if any data from the east coast of Tasmania indicated an elevated biotoxin risk.

### 4.3. PST Analysis

Analyses of all research samples collected prior to 2018 and all risk management samples were conducted via pre-column oxidation HPLC-FLD [35] based on AOAC method 2005.06 [36] at Symbio Laboratories, Sydney; Analytical Services Tasmania; or Cawthron Institute, New Zealand. Research lobster hepatopancreas samples from 2018 on were analysed via LCMS/MS [12,13] at Cawthron Institute, New Zealand. Total PST concentrations from all analysis are expressed in μg or mg STX equiv. kg^−1^, calculated using the Food and Agriculture Organisation/World Health Organisation’s toxicity equivalency factors [37].

Analysis of PST via AOAC method 2005.06 consisted of the extraction of PST from the sample in 1% acetic acid in a boiling water bath for 5 min, followed by cooling, centrifuging, dilution with water, pH adjustment, and SPE cleanup. Aliquots of this extract then underwent periodate oxidation prior to HPLC-FLD analysis to determine the PST screen result. The PST screen typically overestimates sample toxicity as it is assumed that chromatographic peaks generated from co-eluting oxidation products are due entirely to the more toxic analogue (a more detailed explanation of this approach can be found in Harwood et al. [35]). Several PST analogues are known to give multiple co-eluting fluorescent oxidation products. If proceeding to PST confirmation, the initial pH-adjusted extract was separated into 3 fractions via anion exchange SPE. These were then oxidized using both periodate and peroxide, with all of the resulting fractions analysed by HPLC-FLD and the resulting spectra interpreted. PST present in samples were quantified by comparison with certified reference materials. Recovery, dilution, and toxicity equivalency factors were applied to give the contribution of each analogue to sample toxicity. These were then summed to give total sample toxicity in STX equivalents.

Analysis of PST via LCMS/MS occurred as described in Turnbull et al. [8]. PST extraction occurred in 1% acetic acid in a boiling water bath for 5 min, followed by cooling and centrifugation to remove particulate matter. Different ratios of sample to acetic acid were used in the different laboratories. For shellfish, ratios were 2 g in 18 mL, 5 g in 10 mL, or 5 g in 5 mL; for lobster hepatopancreas, the ratios were 2 g in 18 mL or 5 g in 10 mL. This was followed by the addition of ammonium hydroxide before manual SPE cleanup using pre-conditioned amorphous graphitised polymer carbon Supelco ENVI-Carb 250 mg/3 mL cartridges. PST were then eluted with acetonitrile/water/acetic acid (20:80:1 *v*/*v*/*v*) and the eluent diluted with acetonitrile. Sample analysis used HILIC-MS/MS as described by Boundy et al. (2015) and Turner et al. (2015).

### 4.4. Statistical Analysis

All data analyses were performed using the RStudio software (R Core Development Team, version 3.3.6, RStudio, Boston, MA, USA, 2020). All PST results below the level of reporting were designated 0.1 mg STX equiv. kg^−1^ to enable statistical analyses to be performed. Bloom years were designated as 1 May to 30 April as *A. catenella* blooms on the east coast of Tasmania tend to develop in June/July and disperse by December/January. PST results could not be transformed to a normal distribution so the impact of sex and size on PST concentration was assessed using two-way randomized permutation on the 2017–2019 data only, as some hepatopancreas samples collected prior to 2017 contained ovaries. Statistical analyses were assessed against a significance level of 0.05.

To determine the number of lobsters required to be sampled at the start and end of a bloom to give a 95% confidence that the population is below the bivalve ML, all sampling occasions where all lobster were <1.0 mg STX equiv. kg^−1^ were examined. Data from both the start and end of bloom periods were tested for normality and homogeneity of PST concentrations using the Shapiro–Wilk test and Levene’s test. The total PST from the two data sets were not significantly different (*p* value 0.07) and were therefore combined for analysis. A linear model of the mean and the maximum PST concentration was created, and the upper 95% prediction interval limit interpolated to find the mean and maximum total PST concentrations when 97.5% of the population was below the ML.

Only events with four or more consecutive sampling events were used to calculate the uptake and depuration rates of total PST in lobster hepatopancreas. This only occurred for sampling in the Maria zone during 2015 and 2019 (uptake) and 2012, 2017, and 2019 (depuration). The rates of change in total PST were calculated by fitting linear models to the log PST data. Mussel PST concentrations from the same bloom period were visually assessed to determine the appropriate time frame for the associated uptake and depuration of toxins in mussels. Uptake and depuration rates for mussels were then similarly calculated.

## Figures and Tables

**Table 1:** Uptake and depuration rates (exponential) with associated residual variances of total PST in *J. edwardsii* and *M. galloprovincialis* samples in the Maria zone, 2012–2020. Number of samples (*n*) for *J. edwardsii* represents five replicates at each time point, *n* for *M. galloprovinicalis* represents one pooled sample at each time point.

Lobster Site & Year	Phase	*J. edwardsii*		*M. galloprovinicalis* in Spring Bay	
		Rate (mg STX. eFquiv. kg^−1^ day^−1^) (*n*)	Residual Variance	Rate (mg STX equiv. kg^−1^ day^−1^) (*n*)	Residual Variance
Maria Island 2015/16	Uptake	0.009 (20)	0.66	0.075(9)	0.33
Okehampton 2019/20	Uptake	0.019 (20)	1.16	0.032 (12)	0.24
Maria Island 2012/13	Depuration	−0.031 (16)	0.99	−0.043 (12)	0.26
Maria Island 2017/18	Depuration	−0.028 (25)	0.52	−0.072 (23) *	0.52
Okehampton 2019/20	Depuration	−0.017 (20)	1.20	−0.103 (7)	0.17

* Depuration rates significantly different between lobster and mussels, *p* value < 0.0001.

## Data Availability

3rd party data. Restrictions apply to the availability of these data. Data was obtained from the Tasmanian Shellfish Market Access Program, Southern Rock Lobster Limited and the Fisheries Research and Development Corporation and are available from the authors with the permission of these parties.

## Supplementary Materials

The following are available online at https://www.mdpi.com/article/10.3390/md19090510/s1, Table S1: Summary statistics for PST concentration (mg STX.2HCl equiv. kg^−1^) in the hepatopancreas of each treatment group of Southern Rock Lobster *Jasus edwardsii*.
