# Supplementary material for: Field Validation of the Southern Rock Lobster Paralytic Shellfish Toxin Monitoring Program in Tasmania, Australia
Source: Mar Drugs. 2021 Sep 8;19(9):510. doi: 10.3390/md19090510 (PMC8468395; doi:10.3390/md19090510)
Supplement: Supplementary file 1 [file marinedrugs-19-00510-s001.zip › marinedrugs-1352658-supplementary.pdf]

Supplementary Table S1. Paralytic shellfish toxin analogues in *Jasus edwardsii* hepatopancreas from Okehampton Bay, Maria zone, during uptake and depuration in 2019. All concentrations in millimoles kg^{-1}, ND = not detected.

Date	STX	NEO	GTX1&4	GTX2&3	GTX5	C1&2	C3&4	dcSTX	dcGTX2&3	Total
1/07/2019	ND	ND	ND	0.34	ND	ND	0.20	ND	ND	0.54
1/07/2019	ND	ND	ND	0.30	ND	ND	0.20	ND	ND	0.50
1/07/2019	ND	ND	ND	ND	ND	ND	ND	ND	ND	ND
1/07/2019	ND	ND	ND	ND	ND	ND	ND	ND	ND	ND
1/07/2019	ND	ND	ND	ND	ND	ND	ND	ND	ND	ND
5/08/2019	0.43	ND	ND	0.42	ND	2.31	0.61	ND	ND	3.78
5/08/2019	ND	ND	ND	ND	ND	4.42	1.63	ND	ND	6.05
5/08/2019	ND	ND	ND	ND	ND	1.26	0.20	ND	ND	1.47
5/08/2019	ND	ND	ND	0.21	ND	1.68	ND	ND	ND	1.89
5/08/2019	0.30	ND	ND	ND	ND	1.26	ND	ND	ND	1.56
3/09/2019	ND	ND	0.39	0.67	ND	1.89	0.20	ND	ND	3.16
3/09/2019	ND	ND	ND	0.25	ND	0.63	0.20	ND	ND	1.09
3/09/2019	ND	ND	ND	0.25	ND	1.05	ND	ND	ND	1.30
3/09/2019	ND	ND	0.15	0.72	ND	1.26	0.20	ND	ND	2.33
3/09/2019	0.20	ND	ND	ND	ND	ND	ND	ND	ND	0.20
7/10/2019	0.20	ND	1.48	2.49	ND	2.94	ND	ND	ND	7.12
7/10/2019	ND	ND	4.18	5.94	ND	6.94	ND	ND	ND	17.07
7/10/2019	0.27	ND	8.60	9.86	ND	12.41	0.20	ND	ND	31.35
7/10/2019	ND	ND	ND	ND	ND	ND	ND	ND	ND	ND
7/10/2019	ND	ND	ND	ND	ND	ND	ND	ND	ND	ND
22/10/2019	0.17	ND	0.92	1.43	ND	1.89	ND	ND	ND	4.42
22/10/2019	ND	ND	ND	ND	ND	ND	ND	ND	ND	ND
22/10/2019	ND	ND	0.49	0.30	ND	0.63	ND	ND	ND	1.41
22/10/2019	0.40	ND	0.92	3.33	ND	3.37	ND	ND	ND	8.02
22/10/2019	ND	ND	0.12	1.05	ND	1.05	ND	ND	ND	2.23
4/11/2019	0.17	ND	ND	0.59	ND	ND	ND	ND	ND	0.76
4/11/2019	0.23	ND	0.15	1.18	ND	1.05	ND	ND	ND	2.61
4/11/2019	0.23	ND	ND	0.84	ND	0.84	ND	ND	ND	1.92
4/11/2019	0.33	ND	0.19	2.23	ND	1.68	ND	ND	ND	4.45
4/11/2019	0.30	ND	ND	0.97	ND	0.63	ND	ND	ND	1.90
18/11/2019	0.37	ND	ND	1.18	ND	1.26	ND	ND	ND	2.81
18/11/2019	0.43	ND	ND	0.63	ND	0.63	ND	ND	ND	1.70
18/11/2019	0.47	ND	ND	0.34	ND	ND	ND	ND	ND	0.80
18/11/2019	0.37	ND	ND	0.72	ND	ND	ND	ND	ND	1.08
18/11/2019	0.20	ND	ND	0.46	ND	ND	ND	ND	ND	0.66
